# Platelet-Derived Components for Skin and Bone Aging and Age-Associated Pathologies: Mechanisms, Bioengineering Strategies, and Clinical Translation

**DOI:** 10.3390/molecules31050867

**Published:** 2026-03-05

**Authors:** Yuting Liu, Yibin Zheng, Junshan Lan, Qian Huang, Jiayi Chen, Yu Long, Xing Zhou, Ting Zhou, Gang Xiang, Jie Lou

**Affiliations:** 1 School of Pharmacy and Bioengineering, Chongqing University of Technology, Chongqing 400054, China; ytliu@stu.cqut.edu.cn (Y.L.); zhengyobi@stu.cqut.edu.cn (Y.Z.); lanjunshan@stu.cqut.edu.cn (J.L.); huangqian@stu.cqut.edu.cn (Q.H.); chenjiayi309@stu.cqut.edu.cn (J.C.); 12310050122ly@stu.cqut.edu.cn (Y.L.); 2 Yunnan Key Laboratory of Stem Cell and Regenerative Medicine, School of Rehabilitation, Kunming Medical University, Kunming 650500, China; zhouxing@kmmu.edu.cn; 3 Department of Neurology, Xinqiao Hospital, The Army Medical University (Third Military Medical University), Chongqing 400037, China; tingzhou@hospital.cqmu.edu.cn

**Keywords:** platelets, platelet-derived components, skin aging, bone aging

## Abstract

Advances in regenerative medicine have positioned platelets and their derivatives—including platelet-rich plasma, platelet-rich fibrin, platelet lysate, extracellular vesicles, and purified growth factors—as promising interventions specifically for skin and bone aging, two clinically accessible tissues with robust preclinical and clinical evidence for platelet-derived component-based rejuvenation and regeneration. Because much of the available evidence comes from injury models or age-associated inflammatory/degenerative diseases, we explicitly distinguish pathology-targeted inflammation resolution/repair from rejuvenation under physiological aging. This review summarizes the composition and core bioactivities of platelet-derived products and delineates their putative anti-aging mechanisms, encompassing proangiogenic signaling, immunomodulation, attenuation of oxidative stress, regulation of extracellular matrix turnover, and stimulation of osteogenesis. We further evaluate emerging applications that expand therapeutic performance, such as platelet-mimetic delivery vehicles, engineered and sustained-release formulations, and targeted use of subcellular structures. Evidence from recent preclinical and clinical studies indicates favorable safety profiles and signals of efficacy across cutaneous rejuvenation and skeletal regeneration, while underscoring persistent challenges related to product standardization, dosing, and outcome measures. Collectively, platelet-based therapeutics represent a versatile platform with broad applicability to anti-aging interventions in skin and bone and strong potential for translation through continued bioengineering and clinical validation. However, because most available evidence comes from injury models or age-associated diseases (e.g., photoaging, chronic wounds, osteoarthritis, osteoporosis), direct extrapolation to physiological aging is limited; throughout, we explicitly contrast these contexts, specify their indication-specific endpoints, and summarize the main translational limitations.

## 1. Introduction

Population aging is reshaping global health priorities and accelerating the search for safe, effective anti-aging interventions. By 2030, one in six people will be ≥60 years old, rising to 22% by 2050 [1]. Aging (senescence) is characterized by progressive loss of physiological homeostasis, diminished resilience to stress, and cumulative tissue degeneration [2,3]. Clinically, its manifestations include cutaneous aging [4], reduced bone mass and quality [5], sensory decline [6], cognitive impairment [7], and cardiovascular dysfunction [8].

Skin and bone are highly visible and functionally critical targets across the aging spectrum. Here, we distinguish physiological (intrinsic) tissue aging from age-associated pathological processes. Intrinsic cutaneous aging is a natural process characterized by gradual epidermal thinning, dermal collagen and elastin fragmentation, reduced barrier repair, and altered fibroblast/keratinocyte function [9,10]. Low-grade “inflammaging” and oxidative stress can accompany aging, but persistent, high-amplitude inflammation is more typical of extrinsic stressors (e.g., UV-driven photoaging) and inflammatory dermatoses [11,12,13]. In the skeleton, physiological skeletal aging involves progressive imbalance of bone remodeling, loss of bone mass and microarchitecture, and reduced mechanical competence [14,15]. These age-related changes increase vulnerability to diseases such as osteoporosis (OP), a clinical diagnosis defined by fracture risk and/or low bone mineral density; and osteoarthritis (OA), a pathological joint disease in which cartilage degeneration is coupled to synovial inflammation and aberrant subchondral remodeling that drive pain and disability [16,17,18,19].

To ensure scope coherence, we use skin and bone as two exemplar tissues for platelet-derived component (PDC)-based anti-aging. Both are extracellular-matrix-rich tissues in which aging produces quantifiable structural and functional decline—for example, dermal collagen/elastin fragmentation with loss of elasticity, and reduced bone mass, microarchitectural integrity, and mechanical strength—that can be tracked using standardized histologic, imaging, and functional endpoints [20,21]. They are also clinically tractable targets for localized, minimally invasive delivery (e.g., intradermal or intra-articular/intraosseous administration) and longitudinal monitoring, enabling clearer evaluation of formulation, dosing, durability, and safety [22,23]. Finally, compared with many internal organs, the preclinical and clinical evidence base for PDC interventions is relatively mature in cutaneous rejuvenation/wound repair and in bone regeneration/osteoarticular degeneration [24,25], making these tissues appropriate models to synthesize mechanisms, optimization strategies, and translational considerations.

Terminology and scope: throughout this review, we use the terms “skin aging” and “bone/skeletal aging” to denote physiological aging unless otherwise specified. When we discuss photoaging, chronic wounds, OA, OP, or other inflammatory/degenerative conditions, we label them explicitly as age-associated pathologies, because they represent disease processes that may be facilitated by aging but are not synonymous with normal aging. This distinction is important when interpreting mechanisms (e.g., inflammatory cytokines) and evaluating PDC outcomes, which are often measured in disease or injury models. Accordingly, therapeutic benefit observed in inflammatory pathology models (e.g., chronic wounds or OA) should be interpreted as disease-modifying or reparative effects that may improve age-related phenotypes, rather than as direct reversal of normal aging. Because most evidence derives from situations related to injuries or diseases, the Discussion briefly contrasts pathological conditions with physiological aging to clarify context-specific limitations and study-design implications.

As a novel therapeutic modality, PDCs have garnered substantial interest owing to their considerable potential in anti-aging. Comprising platelet-rich plasma (PRP), platelet-derived exosomes (PDEs), and soluble mediators, PDCs are enriched with key growth factors (GFs) such as platelet-derived growth factor (PDGF), transforming growth factor-β (TGF-β), and vascular endothelial growth factor (VEGF). These cues can stimulate fibroblast and endothelial activity, modulate immune responses, enhance angiogenesis and nutrient delivery, and recondition hostile microenvironments. Early studies indicate that intradermal PRP can increase dermal collagen and elastic fiber content and reduce rhytids, whereas PDEs may influence keratinocyte/fibroblast programs and pigmentation via cargo delivery. In bone, PDCs have been reported to support osteoblast genesis, temper osteoclast activity, and help rebalance bone remodeling, suggesting utility for OP and related skeletal decline.

This review first delineates the composition and functional attributes of major PDCs relevant to skin and bone aging, and synthesizes mechanisms of action, including spanning angiogenesis, immunomodulation, oxidative-stress mitigation, extracellular-matrix turnover, and osteogenic/osteoclastic pathways, and aligns them with hallmark processes of aging. The review also critically appraises engineered and functionally modified formulations, highlighting clinical application effects and existing challenges. By linking formulation design to mechanism and indication-specific outcomes, we provide a framework to guide rational selection and optimization of platelet-based interventions for cutaneous and skeletal aging, and to chart actionable paths toward evidence-based clinical adoption.

## 2. Advances in Platelets as Delivery Vehicles

**Figure 1:**
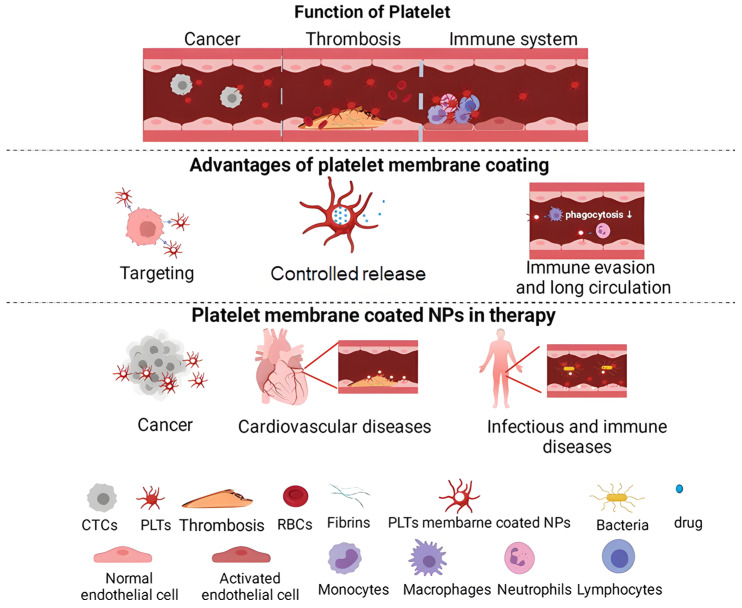
The current development of platelet-membrane-coated nanoparticles in targeted therapy [26]. Adapted from Han, Eur J Pharm Biopharm; published by Elsevier, 2022, under the CC BY 4.0 license, with minor modifications.

Platelets possess distinctive attributes that make them attractive drug-delivery platforms. Table 1 presents the properties of the platelet delivery system compared with those of other delivery vehicles. As endogenous blood components, platelets exhibit excellent biocompatibility and an innate ability to recognize disease-associated microenvironments. For example, platelet-membrane-coated nanoparticles have been applied across multiple indications (Figure 1), illustrating the translational potential of platelet-inspired carriers [26]. Importantly, these delivery principles are also well aligned with anti-aging needs, because aged tissues can develop low-grade inflammaging together with oxidative and proteolytic shifts, whereas age-associated pathologies such as photoaging and OA display more pronounced inflammatory and matrix-degradative niches [27]. Accordingly, platelet-based platforms can be adapted for localized/on-demand delivery of anti-aging cargos (e.g., antioxidants, reactive oxygen species (ROS)-scavenging enzymes/nanozymes, and regenerative peptides), providing a coherent anti-aging rationale for the advantages summarized below [28,29]. Compared with conventional synthetic vectors, platelet-based systems leverage native surface ligands, intracellular storage organelles, and activation programs to achieve targeted, responsive delivery [30]. Key advantages are summarized below. Notably, this ‘homing’ advantage is most relevant when overt injury/inflammation exposes adhesive ligands; in intrinsic aging without clear lesions, localized administration and microenvironment tuning are typically more important than systemic targeting.

**Natural targeting.** Platelets express surface receptors such as P-selectin, glycoprotein VI (GPVI), and glycoprotein Ib α (GPIbα), that enable specific recognition of collagen and von willebrand factor (vWF) exposed at vascular injury sites, as well as adhesion molecules that are overexpressed in inflammatory or tumor microenvironments [31]. This endows platelets with active homing to pathological loci, like GPVI, collagen interactions at atherosclerotic plaques create a “first-stop” interface for payload deposition [32]. In tumors, P-selectin engages cluster of Differentiation 44 on cancer cells, promoting selective platelet accumulation and enabling localized drug delivery [33]. In anti-aging settings, this damage/inflammation tropism can be exploited to enrich cargos in photoaged skin (extracellular-matrix (ECM) exposure, ROS and inflammatory cues) or degenerative osteoarticular niches where matrix breakdown and synovial inflammation facilitate platelet adhesion and payload deposition [34].

**Favorable immunological profile.** Platelets lack major histocompatibility complex class II expression and nuclear deoxyribonucleic acid, reducing the risk of allogeneic immune recognition. Clinically, platelet transfusions often proceed without stringent human leukocyte antigen matching, supporting the feasibility of platelet-derived carriers for scalable applications [35,36].

**High loading capacity and carrier versatility.** The platelet cytoplasm contains abundant α-granules, dense granules, and lysosomes that can be harnessed to encapsulate diverse therapeutic cargos—including anti-oxidative agents (e.g., ROS-scavenging small molecules or polyphenols), regenerative peptides (ECM-restorative or pro-angiogenic peptides), and nucleic-acid therapeutics that modulate senescence-, inflammation-, or matrix-remodeling pathways [28,37,38]—positioning platelets and their membranes as robust biomimetic vectors. In parallel, platelet-derived extracellular vesicles (PEVs; ~40–100 nm) offer efficient drug loading and enhanced tissue penetration relative to many conventional nanocarriers, broadening the delivery toolkit [39].

**Microenvironment-responsive release.** Platelets rapidly activate and release their loaded drugs upon sensing microenvironmental changes, such as elevated ROS, adenosine Triphosphate release, collagen exposure, or expression of specific proteases. Such “on-demand” release is advantageous in thrombosis, metastatic niches, and inflamed tissues. In tumors, for example, matrix metalloproteinases (MMPs) can cleave linkers that tether drug-loaded nanoparticles to platelet membranes, triggering localized drug liberation within the tumor microenvironment [40]. Analogously, protease- and ROS-rich niches characteristic of photoaged skin and osteoarthritic joints can serve as triggers for site-specific release of antioxidants or regenerative peptides, maximizing local efficacy while minimizing systemic exposure [41].

**Table 1:** Comparison of the properties of the platelet delivery system with other delivery vehicles.

Characterizations	Platelet Carrier	Liposome	Erythrocyte Carrier	Polymer Nanoparticles
**Targeting mechanism**	Natural damage targeting	Passive enhanced permeability and retention effect or active modification targeting	Passive delivery, long loops	Active modification targeting
**Immunogenicity**	Extremely low	Medium-high, may induce accelerated blood clearance phenomenon	Low (but blood group restrictions exist)	High (surface finishing required)
**Drug capacity**	High (lumen + membrane bound)	Medium	Medium	High
**Pathological microenvironment responsiveness**	Inherent activation release mechanism	Responsive materials need to be designed	Constraints	Responsive materials need to be designed
**Circulating half-life**	7–10 days	Hours to days	30–60 days	Hours to days
**Difficulty in scaling up production**	Medium (need to optimize freezing techniques)	Low	High	Low
**Refs**	[42]	[43]	[44]	[45]

**Prolonged circulation and potentiated therapeutic activity.** Platelets circulate for approximately 7–10 days, supporting sustained accumulation of loaded drugs at lesion sites [46]. Beyond serving as carriers, platelets actively augment therapy by releasing endogenous bioactive mediators, such as chemokines, messenger RNAs/microRNAs (miRNAs), and GFs, that can act synergistically with the loaded drug. For example, PDEs promote osteogenic differentiation of mesenchymal stem cells during bone repair, platelet-borne miRNAs attenuate inflammatory signaling [47,48], and platelet-derived factors such as insulin-like growth factor 1 (IGF-1) and fibroblast growth factor 2 (FGF-2) modulate local inflammation and stimulate neovascularization during wound healing [49]. This intrinsic drug-biokine synergy, combining exogenous therapeutics with platelet-derived paracrine cues, distinguishes platelet-based delivery systems and offers mechanistically complementary strategies for treating refractory inflammatory and regenerative indications.

## 3. Innovative Applications of Platelet-Derived Components

Platelets hold unique value in skin regenerative medicine, serving not only as a reservoir of bioactive mediators but also as an autologous, intelligent carrier system. Platelet-rich plasma, prepared from peripheral venous blood, concentrates platelets (and their 1100+ bioactive proteins) and typically contains leukocytes [50]. Following activation, platelets release GFs (e.g., VEGF, PDGF, IGF) [51], cytokines, enzymes, and matricellular proteins from α-granules, dense granules, and lysosomes [52]. In parallel, platelet adhesion receptors engage cellular and matrix targets. These soluble and adhesive cues coordinate hemostasis, regulate inflammatory and immune responses, stimulate angiogenesis, and drive cell proliferation and tissue regeneration [53].

**Figure 2:**
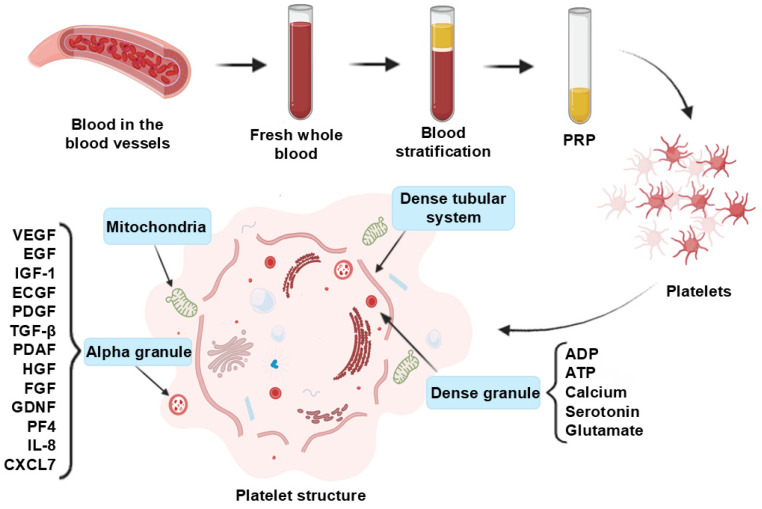
Preparation process and structural components of PRP. Adapted from Vladulescu et al. [55]. Created in BioRender. Huang, Q. (2026) BioRender.com/xqu2dwq.

Leukocyte content is an important modifier of PRP bioactivity. Differences in leukocyte subclasses and progenitors can influence angiogenesis, immunomodulation, intracellular signaling, and metabolic reprogramming [54], thereby shaping repair quality and functional recovery. Through the combined actions of concentrated platelet cargo and leukocytes, PRP establishes an autologous, pro-regenerative microenvironment that supports wound healing. The preparation workflow and structural constituents of dermal PRP are shown in Figure 2.

### 3.1. PRP in Skin Anti-Aging

**Figure 3:**
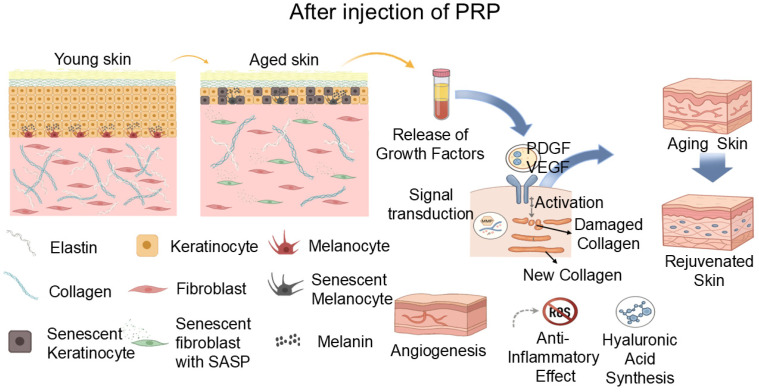
Mechanisms of skin aging and PRP to improve the skin aging process. Adapted from Konstantinou et al. [56]. Created in BioRender. Huang, Q. (2026) BioRender.com/s8tcy9m.

To clarify the physiological processes underlying intrinsic skin aging, Figure 3 depicts a feed-forward network linking cellular senescence, oxidative stress, inflammaging, and ECM remodeling. Chronological aging reduces epidermal stem/progenitor output and keratinocyte turnover, contributing to epidermal thinning and delayed barrier repair [10]. In the dermis, fibroblast senescence and impaired anabolic signaling decrease collagen/elastin synthesis, while ROS-driven stress signaling increases MMP expression and accelerates collagen fragmentation [4,9,56]. Senescent cells and mitochondrial dysfunction amplify low-grade inflammation (“inflammaging”) and inflammasome-associated pathways such as NOD-like receptor family pyrin domain-containing 3 (NLRP3), reinforcing cytokine release and protease activity that further sup-presses ECM homeostasis [11,12,13,56]. These intertwined processes also reduce hyaluronic acid (HA) content and microcirculatory support, manifesting as dryness, laxity, fine wrinkling, and impaired repair capacity [57]. Accordingly, the PRP intervention nodes highlighted in Figure 3 can be interpreted as: restoring growth-factor signaling, rebalancing ECM turnover, supporting angiogenesis, dampening oxidative/inflammatory cascades, and stimulating HA synthesis [56].

In dermatology, many ‘rejuvenation’ datasets are collected in photoaged or otherwise stressed skin, which represents an age-associated pathology with more pronounced inflammation and matrix degradation than intrinsic chronological aging. Current evidence supports five principal modes of action by which PRP exerts its effects: (1) Growth-factor signaling: PRP-derived GFs bind cognate receptors and activate downstream pathways that enhance cellular activity [58], promoting proliferation and migration of epidermal keratinocytes and dermal fibroblasts. (2) Collagen remodeling and synthesis. MMPs present in, or induced by, PRP facilitate clearance of damaged collagen, enabling organized redeposition and improving dermal elasticity with wrinkle reduction [59,60] (3) Angiogenesis and microcirculatory support. GFs like VEGF in PRP stimulate neovascularization [61], enhancing nutrient delivery and waste removal to create a microenvironment conducive to regeneration. (4) Anti-inflammatory and antioxidant effects. PRP can attenuate ROS burden and modulate inflammatory signaling, potentially through regulation of inflammasome activity, e.g., NLRP3 linked to cellular senescence [62,63]. (5) HA induction. PRP stimulates HA synthesis, improving hydration and viscoelastic properties of photoaged skin [57].

Clinical data supports these mechanisms. In split-face and ultraviolet-irradiated skin models, PRP improved texture and reduced rhytids, partly via gene-expression changes [64]. In a prospective study of 30 women, intradermal PRP improved periorbital dark circles, wrinkles, and nasolabial folds [65]. Combination approaches may further enhance outcomes: electroporated PRP-serum showed trends toward improved radiance and firmness [66], and adding botulinum toxin type A to microneedle-delivered PRP improved moisture, elasticity, patient satisfaction, and quality-of-life metrics versus PRP alone [67].

Topical or minimally invasive uses are also promising. A meta-analysis of 19 trials (*n* = 455; 95% female) reported higher patient satisfaction with PRP versus controls for periocular photoaging, with histologic evidence of dermal improvement at 3 months despite moderate heterogeneity (I^2^ = 64%) [68]. In a small series (*n* = 15), PRP improved the features of lip aging without significant adverse effects [69]. Overall, PRP reduces wrinkles, enhances firmness, and improves overall skin quality as a monotherapy or adjunct; however, standardized preparation, dosing, and longer follow-up are needed to confirm durability and optimize topical protocols.

### 3.2. PRP in Skeletal Anti-Aging

Skeletal aging is a multifaceted physiological process involving gradual changes in bone density, microarchitecture, material properties, and overall skeletal resilience. These natural changes can manifest as reduced bone mineral density, altered bone turnover coupling [70], impaired mobility [71], and height loss [72]. Importantly, OP and OA are not inevitable synonyms of “normal” skeletal aging; rather, they are age-associated diseases that arise when age-related vulnerability interacts with additional risk factors and inflammatory or biomechanical drivers. Accordingly, the studies summarized below largely evaluate PRP in OA or OP models, providing evidence for disease modification and tissue repair in pathological microenvironments that commonly coexist with aging. Therefore, when summarizing PRP data in OA or OP, we treat these as evidence of therapeutic activity in pathological microenvironments that often coexist with aging, and we avoid equating them with physiological skeletal aging itself.

**Figure 4:**
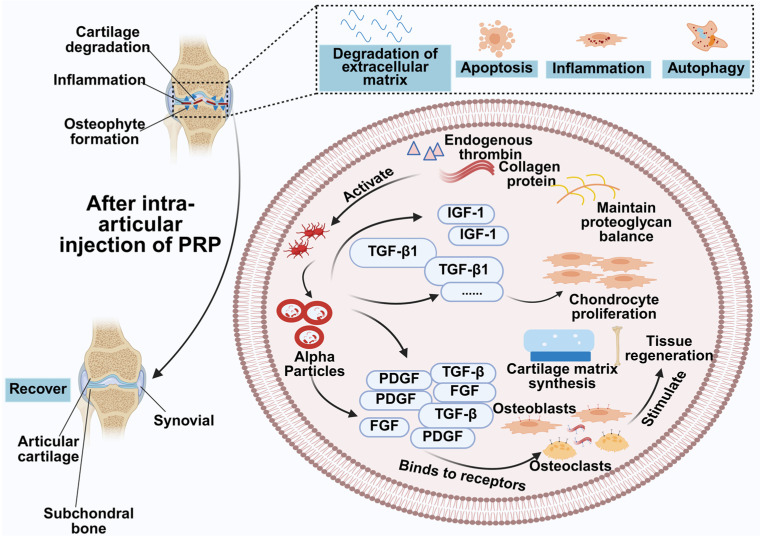
Mechanism of osteoarthritis occurrence, process, and mechanism of PRP treatment of osteoarthritis. Created in BioRender. Huang, Q. (2026) BioRender.com/zvvu55r.

The pathogenesis of OA and PRP’s therapeutic mechanisms are shown in Figure 4. Intra-articular activation of platelets (e.g., by thrombin or collagen) triggers α-granule degranulation and release of PDGF, TGF-β, and FGF [73]. These factors bind receptors on chondrocytes and other joint cells [74], stimulating cartilage-matrix synthesis and repair while exerting anti-catabolic and anti-inflammatory effects: soluble tumor necrosis factor (TNF) receptors can sequester TNF [75]; TGF-β promotes protective secretomes from intra-articular stem cells [76], and upregulates tissue inhibitor of metalloproteinases 1 to inhibit matrix degradation [77]. IGF-1, PDGF, and TGF-β1 further regulate chondrocyte and subchondral-bone metabolism and proteoglycan turnover [78].

Beyond joints, PRP GFs stimulate osteoblast activity and bone-matrix synthesis [79], while VEGF enhances angiogenesis to support repair [80]. In osteoporotic models, PRP upregulates osteogenic genes runt-related transcription factor 2, osteopontin and collagen type Ι alpha 1 chain, promotes mesenchymal stem cell differentiation, and modulates inflammation—reducing interleukin-6, increasing interleukin-10, inhibiting nuclear factor-kappa B, and polarizing macrophages from M1 to M2 phenotypes [81,82,83,84,85].

Studies support PRP’s role in cartilage repair. Platelet-lysate-enriched hydrogels enabled controlled factor release, macrophage recruitment/polarization, and improved cartilage repair [86]. In advanced knee OA, repeated leukocyte-reduced PRP injections reduced pain by 61.6%, with 74% meeting efficacy criteria and magnetic resonance imaging (MRI) improvements in bone-marrow lesions [87]. In another cohort, three intra-articular PRP injections improved visual analogue scale (VAS), knee injury and osteoarthritis outcome score (KOOS) and MRI cartilage characteristics [88]. Combination therapy appears additive: in a randomized study (*n* = 120), PRP + HA achieved a 97.5% response, outperforming either monotherapy and reducing inflammatory markers [89]; a separate analysis suggested better long-term outcomes and fewer adverse events with PRP + HA [90].

For OP, PRP enhanced bone marrow mesenchymal stem cell (BMSC) proliferation/osteogenic differentiation [91], promoted adipose-derived stem-cell proliferation/migration via PDGF/TGF-β signaling [92], and improved functions of the stem cells sorted from periosteum using flow cytometry-sorted periosteal stem cells [93]. PRP-coated 3D-printed titanium scaffolds enhanced bone regeneration in osteoporotic rabbits [94]. In osteoporotic rats, PRP promoted fracture healing via Notch-pathway activation [95], and shockwave-pretreated BMSCs combined with PRP enhanced bone regeneration in rabbit radial defects [96].

Across skin and skeletal indications, PRP acts through convergent, multi-pathway mechanisms—paracrine signaling, immunomodulation, angiogenesis, matrix remodeling, and osteogenic support. While the therapeutic signal is encouraging, progress toward standardized preparation, dosing, delivery, and validated endpoints remain essential to strengthen reproducibility and guide indication-specific protocols.

## 4. Modifying and Engineering Applications of Platelet

To enhance the precision and efficacy of platelet-based drug delivery, recent work has focused on three complementary engineering levers: surface modification, optimization of intraplatelet loading, and microenvironment-responsive release. These approaches preserve platelets’ natural activity while endowing them with new therapeutic functions and targeting capabilities.

### 4.1. Surface Engineered

**Bioorthogonal surface anchoring.** Using acid-labile benzamide linkers, Fan et al. grafted a protein nanogel co-encapsulating the pH-responsive fusogenic peptide glutamic acid-alanine-leucine-alanine (GALA) and granzyme B onto platelet membranes, enabling controlled intracellular protein delivery to prevent postoperative tumor recurrence [97]. Related approaches anchor drug-loaded nanoparticles to platelet membranes via bioorthogonal chemistry or affinity interactions to generate platelet-tumor nanoparticles (PTNPs). Harnessing platelets’ tumor tropism, PTNPs transit to the lesion and, in response to MMPs, release an antiplatelet agent (e.g., ticagrelor), maintaining platelet quiescence, disrupting tumor vasculature, enhancing local drug deposition, and limiting programmed cell death ligand 1 transfer to platelets to alleviate immune suppression [98].

**Cryo-shock membrane opening.** To avoid protein denaturation associated with electroporation, a controlled freeze-thaw (“cryo-shock”) protocol was developed to transiently permeabilize platelet membranes while preserving key receptors (GPVI, GPIbα) for vascular adhesion. The resulting carriers loaded with Mn_3_O_4_ nanoenzymes and a TNF receptor-associated factor 6 inhibitor retained morphology by scanning electron microscope and exhibited fourfold higher collagen adhesion than free nanoparticles (*p* < 0.01; effect abrogated by anti-vWF antibody), supporting targeted therapy for atherosclerosis [99].

### 4.2. Optimization of Internal Drug Loading

**Figure 5:**
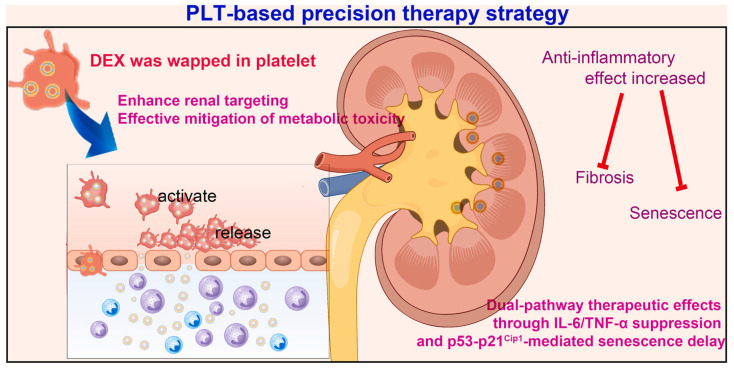
Platelet-based Precision Therapeutic Strategies [100]. Reproduced with permission from Zhang, Bioact Mater; published by Elsevier, 2025.

Transient pore formation can be induced to load small molecules or nanodispersions while maintaining membrane proteins. Using ultrasonic embedding, Zhang et al. achieved high-efficiency loading (≈82%) of dexamethasone lipid nanoemulsions into platelets for renal targeting. Figure 5 illustrates the precision treatment strategy based on platelets. Confocal imaging verified intraplatelet encapsulation; surface markers cluster of Differentiation 41/42b/62P were preserved; and the constructs remained stable for six days at ambient temperature, indicating practical handling and potential clinical utility [100].

### 4.3. Microenvironment-Responsive Release

**Figure 6:**
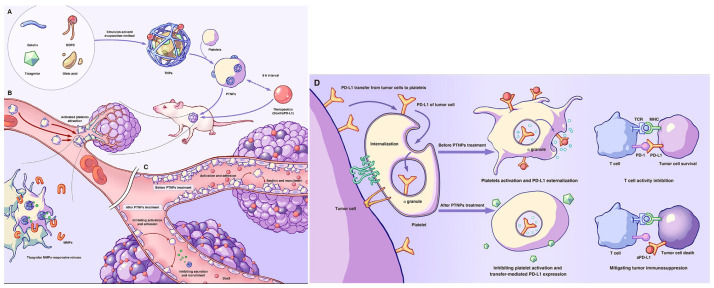
Schematic preparation of PTNPs and proposed action mechanism of PTNPs in vivo. (**A**) Schematic preparation of PTNPs. (**B**) PTNPs could adhere to tumor-associated platelets by specific recruiting ability and responsively release ticagrelor under MMPs stimulation. (**C**) PTNPs inhibited platelets activity, leaded to the leaky tumor endothelial junctions, and promoted the penetration of anti-tumor nanodrugs into tumor tissues. (**D**) PTNPs inhibited platelets activity, reduced transfer-mediated PD-L1 from tumor cells expression on platelets surface, and modulated tumor immunity. Adapted from Lu et al. [40], with cropping and splitting for presentation. Reproduced with permission from Lu, Exploration (Beijing); published by Wiley, 2024.

**Enzyme-triggered delivery.** Elevated MMP-2/9 activity in tumor microenvironments cleaves gelatinous or peptide linkers to unmask or liberate the therapeutic payload. In the PTNPs system, MMP-responsive cleavage at the tumor site releases an antiplatelet agent locally, thereby avoiding systemic platelet inhibition from premature leakage and improving intratumoral drug accumulation [40]. Figure 6 illustrates the preparation process of PTNPs and their potential mechanism of action in the body. Analogously, inflammatory niches enriched for MMP-3/MMP-12 can be exploited for inflammation-targeted release.

**Receptor-independent “hitchhiking.”** To expand the targeting repertoire beyond classical ligand-receptor interactions, Feng et al. designed self-assembling peptide nanofibers that associate with platelets via hydrogen bonding dictated by secondary structure. This receptor-agnostic “hitchhiking” increased lung delivery ~20-fold, offering a generalizable paradigm for vascular bed-specific targeting [101]. Building on this concept, a platelet-hitchhiking biomimetic nanoplatform was engineered to recognize and capture circulating tumor cells, remodel the tumor microenvironment, and suppress recurrence and metastasis [102].

## 5. Application of Platelet Subcellular Components

**Figure 7:**
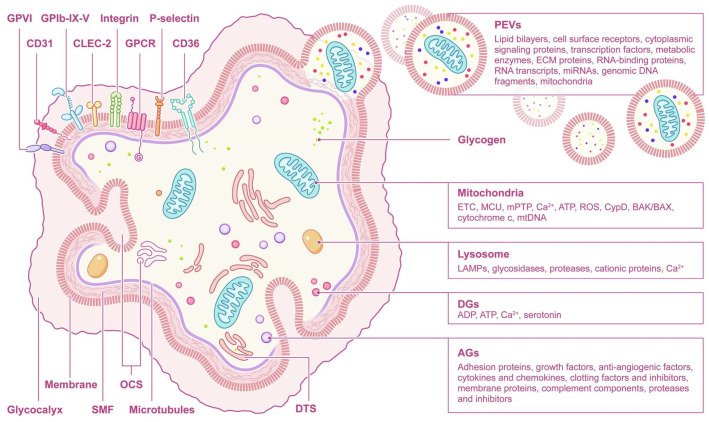
The structure and contents of platelets [103]. Reproduced with permission from Tian, Signal Transduct Target Ther; published by Springer Nature, 2025.

Platelet subcellular components, such as platelet membranes, mitochondria, and exosomes, offer distinct opportunities for regenerative medicine. Figure 7 details the structure and subcellular components of platelets [103]. The platelet membrane is enriched in lipids and signaling proteins that regulate adhesion and activation; for example, vinculin is critical for cytoskeletal coupling and exhibits activation-dependent isoform distribution [104]. Membrane permeability can vary with platelet size and species, a consideration for cross-species modeling and translational design.

**Figure 8:**
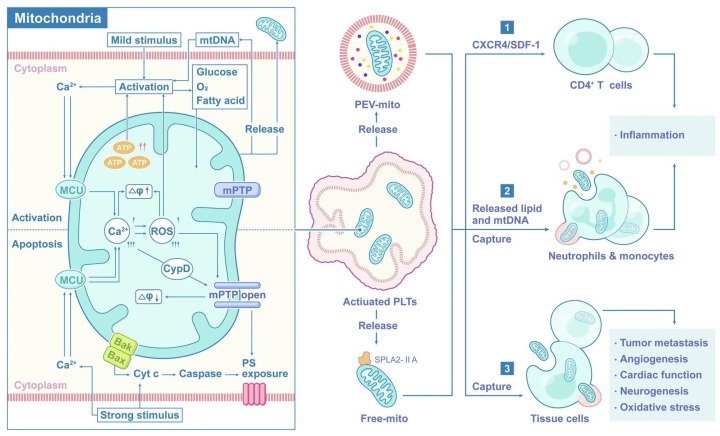
Biological functions of platelet mitochondria [103]. (Arrows in the figure indicate directional changes in biological processes. ↑: Increase in ATP levels or other relevant biological indicators; ↑↑: A more significant or rapid increase in the parameter or process. ↑↑↑: A marked or dramatic increase in the parameter or process, including activation.) Reproduced with permission from Tian, Signal Transduct Target Ther; published by Springer Nature, 2025.

Platelet mitochondria support bioenergetics and redox homeostasis and participate in the regulation of activation and apoptosis [103,105]. Age- or stress-associated mitochondrial dysfunction increases oxidative stress and fosters prothrombotic and proinflammatory states. Excessive mitochondrial excitation can precipitate thrombosis and platelet apoptosis, leading to thrombocytopenia and bleeding. The principal biological roles and regulatory axes of platelet mitochondria are summarized in Figure 8 [103].

Among subcellular products, PEVs are particularly relevant to skin anti-aging [106]. PEVs carry GFs, cytokines, nucleic acids, and lipids that modulate senescence and apoptosis. As natural nanocarriers, PEVs offer excellent biocompatibility and barrier penetration [107]. Common isolation methods include differential ultracentrifugation (high purity but labor-intensive), immunoaffinity capture, freeze-thaw cycling, and sonication. Both hydrophilic and hydrophobic cargos can be loaded—for example, MCC950 (an NLRP3-inflammasome inhibitor) for anti-inflammatory therapy via ultracentrifugation [108] or adriamycin via sonication for tumor targeting [109].

## 6. Application of Platelet-Derived Factors

The biological activity of PRP is closely tied to the GFs concentrated within platelet α-granules [110]. Upon platelet adhesion and activation at injury sites [111,112,113], α-granules release adhesive glycoproteins (e.g., fibronectin, hyaluronan, vWF) and a spectrum of GFs that regulate inflammation, angiogenesis, cell migration, proliferation, and matrix remodeling [114,115]. The biological function of GF is mainly achieved by binding to its specific cell surface receptor, and this binding activates intracellular signal transduction pathways [116], leading to phosphorylation modification of proteins in the cytoplasm on tyrosine residues [117]. Subsequently, protein kinases in the cytoplasm are activated through a cascade phosphorylation reaction and translocate to the nucleus [118]. In the nucleus, these kinases phosphorylate transcription factors, which in turn regulate the transcription process of specific genes [119] and, ultimately, their coding functions.

Table 2 summarizes the Major GFs in PRP and their respective mechanisms of anti-aging function. GFs have been demonstrated to be decisive for the efficacy of PRP [120], including PDGF, VEGF, TGF-β, FGF, IGF, and epidermal growth factor (EGF) [121]. The mechanism of action of these typical GFs is briefly described below.

### 6.1. PDGF

PDGF, a mesenchymal mitogen stored in α-granules, is released in response to tissue injury [128,129]. Four isoforms (PDGF-A, -B, -C, -D) form disulfide-linked homo- or heterodimers that activate PDGFR-α/β, inducing receptor dimerization and multisite phosphorylation [130]. This enables recruitment of SH2-domain adaptors and activation of Ras/MAPK, PI3K, and phospholipase C-γ pathways [131,132,133,134,135]. Downstream effects include mitogenesis, migration, secretion, and phenotypic modulation. PDGF also exerts chemotactic effects on fibroblasts, smooth muscle cells, and neutrophils, thereby supporting re-epithelialization and local anti-inflammatory activity [122].

### 6.2. VEGF

VEGF is a master regulator of angiogenesis with potent endothelial chemotactic and mitogenic actions [136,137]. The biological activity of VEGF is regulated by a variety of factors that include soluble mediators such as cytokines and GFs, as well as cell surface signaling molecules such as CD40 ligand [138]. The function of VEGF is not limited to promoting endothelial cell proliferation and migration [139]; it is also involved in the degradation of ECM [140], vascular maturation, and stabilization processes [123], and acts as a key regulator of endothelial cell survival. In addition, VEGF can induce the expression of a variety of cytokines and chemokines, which can have an important impact on inflammatory responses and immune processes.

### 6.3. TGF-β

TGF-β promotes the differentiation of osteogenic MSCs as well as the proliferation of undifferentiated MSCs [141], while regulating the mitogenic activity of other GFs and inhibiting the proliferation of macrophages and lymphocytes [124]. TGF-β works through the activation of its specific receptor complex, including the activin receptor-like kinase 5 [142], which in turn initiates a series of downstream signaling pathways that are critical for the regulation of MSCs’ proliferation and differentiation. TGF-β not only promotes the regulation of MSCs’ proliferation [143] and differentiation, but also interacts with other GFs such as FGF and PDGF etc. [144], which together constitute a complex regulatory network.

### 6.4. FGF

FGFs regulate tissue repair and organ regeneration and control proliferation/differentiation of osteoblasts, chondrocytes, and MSCs [125,145,146]. Binding to heparan sulfate proteoglycans facilitates FGFR engagement, triggering signaling that promotes cell proliferation and migration [147,148,149]. FGFs support vascular regeneration, limit apoptosis, attenuate injury, and modulate protease expression to drive ECM remodeling and angiogenesis [150,151,152].

### 6.5. IGF

The IGF [153] family regulates a variety of cell biological processes by binding to specific receptors on the cell surface and activating intracellular signal transduction pathways [154]. It is involved in the proliferation and differentiation of chondrocytes [155], with IGF-1 having a key regulatory function in the cartilage formation phase of fracture [156] healing [157]. The binding of IGF-1 to its receptor initiates intracellular cascade signaling, including the activation of the Ras/MAPK signaling pathway [126], and these molecular events promote chondrocyte proliferation and their differentiation to mature chondrocytes. In addition, IGF-1 significantly enhances the synthesis and secretion of cartilage ECM [158], which is essential for the repair and regeneration of cartilage tissue.

### 6.6. EGF

EGF is a class of protein molecules synthesized by cells of the basal layer of the epidermis, which can be secreted by a variety of cell types, including platelets, fibroblasts [159], and macrophages [160]. EGF promotes epithelial regeneration, keratinization, and cell proliferation/migration [127,161]. EGF initiates downstream signaling by binding to the cell surface-specific receptor EGFR, forming a ligand–receptor complex, which in turn activates the receptor’s intrinsic tyrosine kinase [162] activity, followed by a conformational change in the transmembrane structural domains of the receptor and the formation of a stable EGFR homodimer [163]. EGFR also contributes to barrier integrity, inflammation control, and anti-infective responses [164,165].

Platelet-derived modalities, like GF-rich PRP, cytokines, and PEVs, exert anti-aging effects via convergent mechanisms. PRP can mitigate photoaging by engaging the autophagy-NLRP3 axis, improve Ultraviolet B-injured keratinocyte viability, reduce oxidative stress and senescence, and suppress MMP-1 [166]. Topical application of human platelet exosomes remodeled ECM pathways (upregulating collagen/elastin), and reduced senescence signaling and telomere damage [167]. Platelet-derived Prostaglandin E_2_ modulates monocyte/macrophage responses by upregulating interleukin-10 and suppressing TNF after collagen-induced activation [168].

## 7. Discussion and Outlook

### 7.1. Key Translational Barriers

Clinical translation evidence summarized in this review is strongest for PRP in photoaged/stressed skin and in osteoarthritic joints: split-face and UV-irradiated skin studies reported improved texture and reduced rhytids with gene-expression changes [64]; a prospective cohort of 30 women showed improvements in periorbital dark circles/wrinkles and nasolabial folds [65]; adjunct strategies (e.g., electroporated PRP-serum or microneedle PRP plus botulinum toxin type A) further improved radiance/firmness and hydration/elasticity with better patient-reported outcomes than PRP alone [66,67]; a meta-analysis of 19 trials (*n* = 455) found higher satisfaction versus controls with histologic dermal improvement at 3 months despite moderate heterogeneity [68], and a small series (*n* = 15) reported benefits for lip aging without significant adverse effects [69]. For skeletal indications, repeated leukocyte-reduced intra-articular PRP injections in advanced knee OA reduced pain by 61.6% and improved MRI bone-marrow lesions [87], three-injection regimens improved VAS/KOOS and cartilage MRI characteristics [88], and in a randomized study (*n* = 120) PRP + HA achieved higher response than either monotherapy with reduced inflammatory markers and favorable safety [89,90]. Across these studies, PRP appears generally safe and effective as an anti-inflammatory, pro-repair adjunct, but evidence grading is limited by major translational barriers: heterogeneity in PRP/PEVs preparation (platelet/leukocyte content, activation, storage), incomplete reporting of delivered platelet/EV dose and dosing frequency, variable comparators and outcome measures, short/uneven follow-up, and inconsistent adverse-event documentation; addressing these with standardized classification/reporting, dose–response optimization, longer follow-up, and good manufacturing practice-aligned engineered formulations (especially for EVs) is essential for reproducible, indication-specific protocols.

Despite encouraging preclinical and early clinical signals across cutaneous rejuvenation and osteoarticular repair, translation of PDCs into evidence-based anti-aging interventions remains constrained by four interrelated issues: mechanistic heterogeneity, inconsistent safety/protocol reporting, lack of product standardization, and non-uniform outcome measures. Among these, inadequate standardization is the most immediate barrier to reproducibility, cross-study comparison, and dose–response reasoning.

In practical terms, PDCs tend to show the clearest efficacy as anti-inflammatory, pro-repair adjuncts in injury- or disease-enriched microenvironments (e.g., photoaged skin, chronic wounds, OP) [169], where platelet adhesion/activation cues and matrix remodeling signals are abundant. By contrast, physiological (intrinsic) aging is typically characterized by lower-amplitude, chronic ‘inflammaging’ [170] and less overt ECM exposure [171], which may blunt platelet homing and alter the magnitude and kinetics of PDC responses. Therefore, outcomes established in pathology models (pain relief, wound closure, defect filling) should not be over-interpreted as evidence of true rejuvenation; aging-focused studies should prioritize aging-relevant endpoints (barrier function, elasticity, senescence markers, bone microarchitecture/mechanics), assess durability after treatment withdrawal, and systematically monitor fibrosis, aberrant angiogenesis, and thrombosis-related risks, especially under repeated dosing [172].

### 7.2. Standardization: Why PRP Classification Matters

“PRP” is not a single product but a family of preparations that differ [173] substantially in (i) platelet concentration and total injected platelet dose, (ii) leukocyte/neutrophil and red blood cell (RBC) contamination, (iii) fibrin architecture (plasma vs. fibrin matrix), and (iv) activation method (endogenous vs. exogenous; calcium/thrombin, etc.). These variables shift the balance between pro-regenerative signaling and pro-inflammatory/pro-catabolic cues and therefore can materially change clinical outcomes. Consequently, adopting a transparent PRP classification system—reported alongside quantitative cell counts and preparation parameters—should be treated as a minimum requirement for anti-aging studies in both skin and bone. Representative systems include PAW, PLRA, DEPA, and broader reporting frameworks such as MARSPILL and MIBO (Table 3). Using any established system is preferable to an unqualified “PRP,” and combining a classification label with quantitative reporting (including injected volume and total platelet dose) enables meaningful meta-analyses and protocol harmonization across indications.

To make these classifications actionable, anti-aging PRP studies should report baseline and final platelet/white blood cell (including neutrophil fraction)/RBC counts, preparation and activation parameters, injected volume, and the total delivered platelet dose, so that protocols can be compared quantitatively and synthesized across studies.

### 7.3. Outlook

Ultimately, advancing PDCs toward standardized, scalable anti-aging therapeutics will require: (i) mechanistic studies in aging-relevant models that map cell-type-specific targets and signaling networks; (ii) consensus adoption of classification-plus-quantitation reporting to enable reproducibility and pooled analyses; (iii) adequately powered, long-term randomized trials using standardized formulations and validated endpoints; and (iv) health-economic evaluations that incorporate manufacturing costs, visit burden, durability of benefit, and equity of access. Addressing these priorities will clarify where platelet-derived therapies provide durable, clinically meaningful rejuvenation and will accelerate their responsible integration into dermatologic and musculoskeletal practice.

## Figures and Tables

**Table 2:** Major growth factors in PRP and their anti-aging function.

Growth Factors	Main Sources	Target Cells	Core Anti-Aging Functions	Mechanisms of Action	Refs
**PDGF**	Alpha particles, etc.	Fibroblasts, mesenchymal stem cells, etc.	Stimulates collagen synthesis, promotes fibrous tissue regeneration, etc.	Activation of the mitogen-activated protein kinase/extracellular signal-regulated kinase pathway and up-regulation of collagen gene expression	[122]
**VEGF**	Alpha particles, etc.	Vascular endothelial cells, etc.	Promotes neovascularization, improves tissue microcirculation, etc.	Binds VEGFR2 and activates the phosphatidylinositol 3-kinase/protein kinase B (PI3K/Akt) pathway	[123]
**TGF-β**	Alpha granules, platelet membranes, etc.	Fibroblasts, chondrocytes, etc.	Increased extracellular matrix (ECM) deposition, inhibition of metalloproteinases, etc.	Modulation of the Smad signaling pathway to promote fibronectin production	[124]
**FGF**	Alpha particles, etc.	Fibroblasts, keratinocytes, mesenchymal stem cells, etc.	Promotes the proliferation and migration of various cell types and activates tissue stem cells, etc.	Activates downstream signaling pathways such as rat sarcoma virus oncogene homolog/mitogen-activated protein kinase (RAS/MAPK) and PI3K/AKT to promote cell proliferation, survival, migration, and matrix synthesis	[125]
**IGF**	Alpha particles, etc.	Vascular endothelial cells, chondrocytes, etc.	Reduces apoptosis, promotes protein regeneration, etc.	Activation of insulin receptor substrate 1/phosphatidylinositol 3-kinase pathway and inhibition of caspase activity	[126]
**EGF**	Alpha particles, etc.	Keratinocytes, fibroblasts, etc.	Promotes cell regeneration, stimulate collagen synthesis, etc.	Stimulates EGFR and promotes cell migration and proliferation	[127]

**Table 3:** Representative PRP classification and reporting frameworks relevant to standardization.

System (Year)	Key Variables Captured	Strengths	Main Limitations	Refs
**Dohan Ehrenfest classification** **(P-PRP/L-PRP/P-PRF/L-PRF; 2009–2014)**	Leukocytes + fibrin architecture (plasma vs. fibrin matrix)	Clarifies PRP vs. PRF families; useful when fibrin matrix or solid constructs are used	Does not quantify platelet dose; does not separate neutrophil subtypes	[174]
**PAW (2012)**	Platelet concentration; activation method; white blood cell (WBC) (incl. neutrophil category)	Simple, clinically intuitive; enables comparison of cellular content and activation across studies	Platelet ‘dose’ (total injected platelets) still needs explicit reporting	[175]
**PLRA (2015)**	Platelet concentration; leukocyte content; red blood cell (RBC) content; activation	Adds RBC contamination (often underreported) to platelet/leukocyte/activation description	Categories can be coarse; still benefits from absolute counts and injected volume	[176]
**DEPA (2016)**	Dose of injected platelets; efficiency of production; purity (RBC/WBC); activation	Directly links to delivered dose, a key determinant of biological effect; encourages manufacturing transparency	Requires baseline/final counts to compute; adoption varies across fields	[177]
**MARSPILL (2017)**	Method, activation, RBCs, spin, platelet concentration, image-guided injections, leukocytes, light activation	Comprehensive; captures preparation workflow variables that influence product composition	More complex; may reduce adoption if data collection is incomplete	[178]
**MIBO checklist (2017)**	Minimum reporting items for PRP studies (counts, volume, prep details, delivery)	Not a ‘classification’ per se, but a practical standard to ensure reproducibility and comparability	Requires author compliance; needs adaptation for dermatology/anti-aging endpoints	[179]

## Data Availability

No new data were created or analyzed in this study. Data sharing is not applicable to this article.

## Abbreviations

The following abbreviations are used in this manuscript:

BMSC	Bone marrow mesenchymal stem cell
ECM	Extracellular Matrix
EGF	Epidermal growth factor
FGF-2	Fibroblast Growth Factor 2
GALA	Glutamic Acid-Alanine-Leucine-Alanine
GFs	Growth Factors
GPIbα	Glycoprotein Ib α
GPVI	Glycoprotein VI
HA	Hyaluronic acid
IGF-1	Insulin-like Growth Factor 1
KOOS	Knee Injury and Osteoarthritis Outcome Score
miRNAs	microRNAs
MMPs	Matrix metalloproteinases
MRI	Magnetic Resonance Imaging
NLRP3	NOD-like Receptor Family Pyrin Domain-Containing 3
OA	Osteoarthritis
OP	Osteoporosis
PDC	Platelet-Derived Component
PDEs	Platelet-Derived Exosomes
PDGF	Platelet-Derived Growth Factor
PEVs	Platelet-derived extracellular vesicles
PI3K/Akt	Phosphatidylinositol 3-Kinase/Protein Kinase B
PRP	Platelet-Rich Plasma
PTNPs	Platelet-tumor nanoparticles
RAS/MAPK	Rat Sarcoma Virus Oncogene Homolog/Mitogen-Activated Protein Kinase
RBC	Red Blood Cell
ROS	Reactive oxygen species
TGF-β	Transforming Growth Factor-β
TNF	Tumor Necrosis Factor
VAS	Visual Analogue Scale
VEGF	Vascular Endothelial Growth Factor
vWF	von Willebrand factor
WBC	White Blood Cell
